# Dual Roles of Hypoxia-Inducible Factor 1 in Acute Lung Injury: Tissue-Specific Mechanisms and Therapeutic Modulation

**DOI:** 10.3390/cells14141089

**Published:** 2025-07-16

**Authors:** Junjing Jia, Yingyi Zhang, Qianying Lu, Sijia Tian, Yanmei Zhao, Haojun Fan

**Affiliations:** 1 School of Disaster and Emergency Medicine, Tianjin University, Tianjin 300072, China; 2024246222@tju.edu.cn (J.J.); yingyi_zhang@tju.edu.cn (Y.Z.); qianying.lu@tju.edu.cn (Q.L.); 2023246184@tju.edu.cn (S.T.); 2 Tianjin Key Laboratory of Disaster Medicine Technology, Tianjin 300072, China

**Keywords:** acute lung injury, hypoxia, hypoxia-inducible factors, inflammation, tissue repair, regulation

## Abstract

Acute lung injury (ALI), a life-threatening clinical syndrome with multifactorial origins, is characterized by uncontrolled pulmonary inflammation and disrupted alveolar–capillary barrier integrity, leading to progressive hypoxemia and respiratory failure. In this hypoxic setting, hypoxia-inducible factor (HIF)-1 is activated, acting as a central regulator of the inflammatory response and reparative processes in injured lung tissue during ALI. The role of HIF-1 is distinctly dualistic; it promotes both anti-inflammatory and reparative mechanisms to a certain extent, while potentially exacerbating inflammation, thus having a complex impact on disease progression. We explore the latest understanding of the role of hypoxia/HIF-mediated inflammatory and reparative pathways in ALI and consider the potential therapeutic applications of drugs targeting these pathways for the development of innovative treatment strategies. Therefore, this review aims to guide future research and clinical applications by emphasizing HIF-1 as a key therapeutic target for ALI.

## 1. Introduction

The lungs are the primary organ responsible for fulfilling the systemic oxygen demands essential for cellular metabolism [1]. Acute lung injury (ALI), a life-threatening respiratory syndrome with global clinical significance, arises from heterogeneous etiologies involving both endogenous and exogenous insults. Common triggers include bacterial pneumonia, systemic sepsis, polytrauma, and pancreatic necrosis, all of which may progress to acute respiratory distress syndrome (ARDS) through shared pathobiological pathways [2,3]. The pathophysiological hallmark of ALI involves dysregulated pulmonary inflammation and compromised microvascular integrity, which clinically manifests as a triad of (1) oxidative stress amplification, (2) neutrophil-dominated leukocyte infiltration, and (3) protein-rich alveolar edema secondary to endothelial–epithelial barrier disruption [4]. These pathological alterations collectively impair gas exchange efficiency by reducing alveolar surfactant levels and increasing capillary permeability, ultimately culminating in hypoxemic respiratory failure [5]. Notably, both pulmonary and extrapulmonary factors can initiate this cascade by damaging the alveolar–capillary unit—a functional syncytium comprising type I/II pneumocytes and endothelial cells—whose structural integrity is critical for maintaining physiological gas exchange [4,5].

Hypoxia serves as a critical pathophysiological driver in ALI, not only by amplifying systemic inflammatory cascades but also by directly compromising pulmonary endothelial–epithelial homeostasis [6]. The dynamic adaptation of the vasculature to oxygen deprivation represents a sophisticated biological mechanism, with hypoxia-inducible factor 1 and 2 (HIF-1 and HIF-2) isoforms serving as principal regulators of this hypoxic response [7]. HIFs are a family of nuclear transcription factors that are pivotal in adjusting cellular reactions to hypoxic conditions, orchestrating the regulation of a multitude of genes that govern physiological processes such as metabolism, angiogenesis, erythropoiesis, and other hypoxia-associated adaptations [8]. HIF signaling plays a critical role in modulating inflammatory lung injury and repair processes in ALI, with its effects being influenced by specific HIF isoforms [9]. Lung cell types such as lymphatic endothelial cells (LECs) and alveolar epithelial cells (AECs), as well as infiltrating leukocytes, and the nature of the injury stimulus are involved [7].

Despite the array of therapies employed in the clinical management of ALI, including glucocorticoids such as dexamethasone [10], neutrophil elastase inhibitors [11], granulocyte-macrophage stimulator (GM-CSF) [12], statin drugs [13] and inhaled nitric oxide [14], their ability to inhibit pro-inflammatory mediators has been limited. Specifically, the benefits and risks of steroid treatments vary and may exacerbate inflammation [15,16]. Sivelestat, a neutrophil elastase inhibitor associated with short-term improvements in oxygenation, has not been shown to reduce the duration of mechanical ventilation or ALI mortality [17]. GM-CSF, with its potential protective effects on AECs and influence on the progression to fibrosis [18], may also contribute to multiorgan failure and systemic inflammation [12]. Statins have shown no reduction in in-hospital mortality [19], and while inhaled nitric oxide improves oxygenation and pulmonary endothelial function [14], it does not decrease ALI mortality [20]. Mechanical ventilation, which is common for hypoxic patients, can further aggravate lung injury [21], with elevated airway pressures and larger tidal volumes contributing to a higher incidence of ALI/ARDS [22]. Understanding the mechanisms underlying damage and identifying new targets for targeted therapy is therefore beneficial in terms of palliation and curing the disease. Given that the lungs constitute the primary organ for gas exchange in mammalian systems, diminished oxygen tension coupled with elevated carbon dioxide accumulation is a central pathophysiological hallmark of ALI, and insufficient oxygen leads to a range of inflammatory diseases [23]. HIF-1 functions as a central transcriptional regulator orchestrating cellular adaptation to hypoxia amidst inflammatory conditions [24], and it can coordinate the transcriptional program and regulate metabolism [23]. HIF-1 plays an extremely complex and multifaceted regulatory role in ALI and subsequent tissue repair.

In this review, we describe a systematic analysis of HIF-1 expression in ALI. We highlight the critical regulation of the inflammatory response by HIF-1 and its significant impact on alveolar epithelial and endothelial cells. Additionally, we examined the role of HIF-1 in angiogenesis, alveolar epithelial regeneration, and extracellular matrix remodeling, which are essential for tissue repair. The principal focus of this study was to analyze the therapeutic role of HIF-1 in enhancing clinical outcomes and promoting tissue regeneration in ALI. This assessment aims to guide future research and clinical applications, emphasizing the role of HIF-1 as a key target for advancing ALI treatment strategies.

## 2. Pathophysiology of ALI

**Figure 1:**
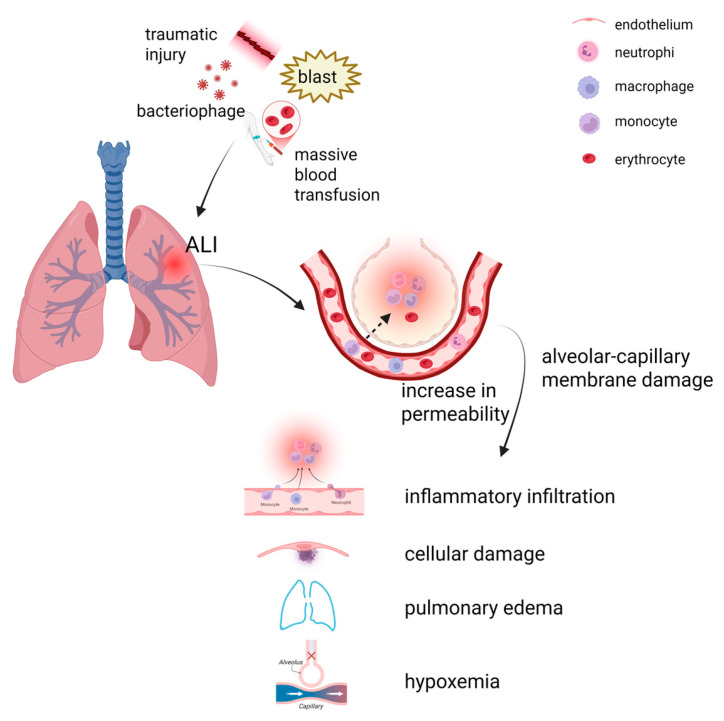
Pathophysiology of ALI. Direct and indirect factors such as pneumonia and concussion lead to ALI, causing impairment of the pulmonary epithelial-endothelial barrier and infiltration of inflammatory cells, such as neutrophils, into the alveoli, which promotes the progression of ALI. Created in BioRender. https://BioRender.com.

ALI is a series of pulmonary changes triggered by diverse pulmonary insults, that frequently contribute to significant clinical complications and elevated mortality rates [25]. There are many causes of ALI, including a variety of direct and indirect causative factors. Lung injuries (direct factors) include severe alveolar lesions caused by severe lung infections, pulmonary contusions, pulmonary embolism, inhalation of toxic gases, drowning, trauma and so on; Non-pulmonary injuries (indirect factors) include various organ injuries, massive blood transfusions, extracorporeal circulation, pancreatitis and the drug overdose, which first trigger uncontrolled systemic inflammation and ultimately lead to vascular endothelial damage and multiple inflammatory cell infiltrates [26]. These direct and indirect factors can lead to a series of physiological responses secondary to alveolar–capillary membrane damage, which can cause increased permeability, alveolar leakage of protein-rich fluid and microthrombosis, and lead to cellular damage, acute hypoxic respiratory insufficiency, and alveolar edema (Figure 1). The clinical manifestations are progressive hypoxemia and respiratory distress. These secondary reactions may further exacerbate changes in the pulmonary microenvironment. Without timely management intervention, ALI can further worsen, leading to alveolar effusion and atrophy of most alveoli, preventing oxygen from entering the bloodstream from inhaled air and leading to a dramatic drop in blood oxygen levels, which may lead to ARDS. Owing to lung damage and the inflammatory response, patients with ARDS have a weaker ability to fight lung infections and are susceptible to bacterial pneumonia, which further exacerbates their condition, thus ALI/ARDS usually results in massive respiratory failure and death, with in-hospital deaths of up to 38–46% [27].

### 2.1. Direct Factors

Pneumonia, a common predisposing factor for ALI, is clinically significant because of its pathogenicity. Once lower respiratory tract infections, involve the lung parenchyma, they often arise from viral-bacterial copathogenicity [28]. The combination of viral invasion, which directly damages the cells of the lung tissue, and localized bacterial proliferation and the release of toxins, results in severe damage to the alveolar barrier. This destruction results in abnormal changes in the permeability of the alveolar capillaries, and large amounts of fluids, proteins, neutrophils, and erythrocytes leak out, which allows for a large accumulation of these substances in the alveolar lumen [29], resulting in excessive pulmonary effusion [30], which can lead to inflammation of the lungs. The main etiology of ALI is pneumonia, and its pathophysiological process involves a complex mechanism of action involving inflammatory cells. Inflammatory cells are attracted to neutrophil aggregates in the lungs by inflammatory chemokines, and neutrophils are activated after arriving in the pulmonary vasculature. Activated neutrophils release a series of toxic substances, including oxygen free radicals, elastase, collagenase, and other [31] toxic substances, which damage the alveolar-structure and function of the capillary membrane.

Lung trauma constitutes another common causative factor in ALI, where the mechanical force itself acts directly on the lung tissue, resulting in structural damage and injury, while at the same time the body’s inflammatory response mechanisms are rapidly activated. Local signals of tissue damage induced by trauma prompt inflammatory cells to converge towards the site of injury, ultimately leading to increased leakage of alveolar capillary permeability [32]. Traumatic lung injury tends to be more extensive, and involves multiple structural levels of lung tissue, such as alveoli, pulmonary vasculature, and interstitial tissue. In addition to the intertwining of mechanical injury and the inflammatory response, there may be complications such as hemorrhage, pneumothorax and hemothorax, which further exacerbate the degree of damage to and dysfunction of the lung tissue. Traumatic lung injury therefore presents a more severe and complex pathology than other types of lung injury do and is more likely to progress to respiratory distress syndrome [33].

### 2.2. Indirect Factors

In addition to pneumonia, lung trauma and other factors that can directly trigger ALI, other organ injuries can also induce ALI. In particular, ALI is more common after traumatic brain injury (TBI), a complication that can progress to ARDS, with a mortality rate ranging from 30 to 40% [34]. TBI can cause lung injury via the neuro-respiratory inflammatory axis by increasing immune reactivity in the lungs [34]. Specifically, after the onset of TBI, there are stressful changes in the body’s neuroendocrine system, with sympathetic excitation and the release of large amounts of neurotransmitters. These neurotransmitters can act on the pulmonary vasculature, causing changes in local perfusion on the one hand and inducing redistribution of microthrombi within the pulmonary vasculature on the other hand. This redistribution of microthrombi, as well as local perfusion abnormalities, leads to enlarged alveolar cavities. Moreover, lung surfactant is overstimulated in this process, which in turn activates sympathetic nerves. Excessive sympathetic activation can cause severe disruption of the mechanisms regulating the balance of ventilation and perfusion in the lungs, resulting in impaired gas exchange and hypoxemia. Microthrombi redistribution within the pulmonary vasculature can impair local blood flow and enlarge alveolar spaces. This structural disruption may overstimulate sympathetic nerves, potentially due to altered pulmonary surfactant activity, and destabilize the ventilation-perfusion balance. The resulting hypoxemia can trigger a surge of unstable zinc and inflammatory mediators in the lungs, exacerbating tissue damage [35]. This further promotes cascade amplification of the inflammatory response, exacerbating the extent of lung tissue damage and driving the continuation of ALI.

In addition, while clinical blood transfusions effectively replenish blood components and improve hematological parameters, they also carry risks. Among these complications, transfusion-associated ALI is among the most severe outcomes. The transfusion of anti-leukocyte antibodies or biological response modifiers (BRMs) during or after blood transfusion can also lead to ALI. Transfused anti-leukocyte antibodies bind to leukocyte surface antigens in the recipient’s blood, whereas BRMs act directly on immune cells, activating immune cell populations such as monocytes and neutrophils, which release reactive oxygen species, damage the lung endothelium and cause fluid leakage [36]. As fluid leakage continues, the fluid content of the lung tissue gradually increases, eventually leading to the development of pulmonary infiltrates. This pulmonary infiltrate is characterized by hypoxia and non-cardiogenic pulmonary edema as its main clinical feature [37].

### 2.3. Endothelial and Epithelial Cell Apoptosis, and Neutrophil Recruitment

ALI, particularly its most severe manifestation, ARDS, is a life-threatening condition characterized by elevated mortality rates and a pathology characterized by endothelial dysfunction, lung epithelial damage and accumulation of neutrophils [38,39]. Endothelial cells are located in the inner wall of blood vessels, and as an important part of the inner wall of blood vessels, they play a multifaceted and critical coordinating role in maintaining the normal physiological function of blood vessels. The coordination of many physiological functions, such as the regulation of blood flow, vascular tone, and cell and nutrient transport under normal conditions, promotes the formation of new blood vessels. Endothelial cell dysfunction is usually associated with disruption of the endothelial barrier, which makes it easier for intravascular fluids, proteins, and cellular components to leak into the alveolar compartment, triggering an exaggerated inflammatory cascade that subsequently induces elevated permeability of the pulmonary vasculature, leading to alveolar flooding and substantial neutrophil accumulation within alveolar spaces [40]. Alveolar flooding and neutrophil infiltration interact with each other, further destroying the normal structure and function of the alveoli and facilitating the development of pulmonary edema [41].

In the complex pathophysiological process of ALI, epithelial cells play a crucial role. The alveolar epithelium is predominantly composed of two specialized epithelial cell types, classified as type I and type II. Alveolar type I epithelial (ATI) cells play a key role in gas exchange between alveoli and capillaries, and alveolar type II epithelial (ATII) cells secrete alveolar surfactant [42]. Its regulation of surfactant metabolism to maintains alveolar function. Diffuse alveolar epithelial damage attacks and destroys alveolar structures directly or indirectly during the onset and progression of ALI/ARDS by activating a pattern of damage-associated molecules (DAMPs) released by macrophages and neutrophils [43]. Once released into the alveolar microenvironment, these DAMPs act as danger signals, which are detected by pattern recognition receptors (PRRs) located on the membranes of various immune cells. This recognition process further activates downstream signaling pathways, thereby triggering a more widespread and intense inflammatory response. A substantial population of inflammatory cells are recruited to the alveolar region, where they release more inflammatory mediators and create an inflammatory cascade amplification effect. Thus lung epithelial cell dysfunction is considered an important factor contributing to ALI progression. ATII cells are uniquely equipped to synthesize and secrete lung surfactants, and damage to ATII cells results in a decrease in their synthesis and secretion of lung surfactants. Lung surface-active substances are complex mixtures of lipids and proteins whose central role is to effectively reduce alveolar surface tension. Under normal physiological conditions, appropriate alveolar surface tension ensures stable alveolar morphology and function during respiration, and prevents the occurrence of alveolar atrophy [44]. When it decreases, the surface tension of the alveoli increases, and the higher surface tension makes it difficult to maintain normal expansion of the alveoli at the end of exhalation, and the alveoli tend to atrophy. The presence of alveolar atrophy leads directly to atelectasis, which destabilizes the alveolar barrier. The alveolar barrier is an important structural basis for gas exchange between alveoli and capillaries and for maintaining fluid balance in the lungs. Its unstable state not only weakens the efficiency of gas exchange and severely affects the ventilatory function of the lungs, but also disrupts the homeostatic balance of the microenvironment in the lungs, thus promoting the accumulation of alveoli and the migration of inflammatory cells and protein-rich fluids and other macromolecules into the alveolar lumen [27]. Rapid amplification of the inflammatory response and exacerbation of ALI/ARDS.

In the pathophysiological course of ALI and ARDS, endothelial and epithelial cell damage frequently coincides with neutrophil infiltration into the interstitial and bronchoalveolar spaces. Neutrophils, important components of the innate immune response, play pivotal roles in the initiation of the immune-inflammatory response in ALI/ARDS and are among the earliest immune cell types recruited to the site of lung tissue injury. Upon activation, neutrophils are able to efflux from the vascular system and migrate through the interstitial tissue into the alveolar lumen [45]. In addition to neutrophil extravasation, increased neutrophil activation contributes to collateral tissue damage and alveolar–capillary barrier breakdown during ALI/ARDS [46]. Excessive pro-inflammatory cytokines are released by activated neutrophils and alveolar macrophages [47], such as tumor necrosis factor-alpha (TNF-α), interleukin 6 (IL-6) and interleukin-1β (IL-1β). These pro-inflammatory cytokines accumulate rapidly locally in lung tissue, forming a strong inflammatory signaling network that further recruits more immune cells to the site of injury, thus significantly increasing the intensity and extent of the inflammatory response and tissue damage during ALI [48].

### 2.4. Polarization of Macrophages

Alveolar macrophages (AMs) play crucial roles in the immune defense system of lung tissue. It is widely distributed in the lumen of airway interstitial spaces close to the lung epithelium, and acts as the first line of lung immune defense on the surfaces of large and small airways and alveoli [49], and it is the most important immune cell in the lung tissue, with roles as professional removal of cellular debris and promotion of inflammation resolution [2]. As major effector cells in the immune response process, macrophages possess unique dual pro-inflammatory and anti-inflammatory properties [50]. In response to various external stimuli, macrophages can differentiate into different functional phenotypes on the basis of the real-time state of the microenvironment and its dynamics, with pro-inflammatory (M1) macrophages, which are classically activated and selectively activated or anti-inflammatory (M2) macrophages, which are the most typical types [51]. The M1 phenotype induces the production of pro-inflammatory Th1 cytokines (e.g., IFN-γ and LPS) and high levels of pro-inflammatory factors, including IL-1β, IL-12, TNF-α and inducible nitric oxide synthase (iNOS). The M2 phenotype can involve the induction of the Th2 cytokines IL-4 and IL-13 and the production of anti-inflammatory molecules such as IL-10 [52]. It has the capacity to suppress the production and secretion of pro-inflammatory cytokines while modulating immune cell functionality, thereby attenuating the intensity of the inflammatory response and fostering both tissue repair/regeneration and inflammation resolution. This plasticity of macrophage phenotypic differentiation and the different functional effects it produces are crucial for maintaining immune homeostasis in lung tissue and for coping with various lung diseases.

Macrophages are involved in the progression of ALI/ARDS and are key factors in controlling inflammatory processes and facilitating the regeneration of impaired lung tissue [52]. After the lungs are subjected to bacterial or traumatic stimuli, the homeostasis of the internal environment is disrupted, and a series of complex changes occur in the local microenvironment of the lungs. Driven by these changes, macrophages differentiate and accumulate predominantly towards the M1 phenotype during the exudative phase of the acute inflammatory response in ALI/ARDS. M1 macrophages enter the lungs and alveoli from the circulation by secreting a variety of pro-inflammatory cytokines and recruiting neutrophils [53], which in turn leads to further inflammation and exacerbation of lung injury.

### 2.5. Vascular Leakage and Hypoxemia

Lungs, as organs of gas exchange, are highly susceptible to lung tissue damage under hypoxia, which manifests itself as pulmonary hypertension and increased permeability of pulmonary capillary endothelial cells, leading to a large amount of fluid entering the interstitial cavities of the lungs, and ultimately leading to edema and ALI [54]. Fluid accumulation in the alveoli reduces the effectiveness of air exchange between the alveolar and vascular systems, leading to hypoxemia and localized alveolar hypoxia [55]. A major pathological feature of ALI is local alveolar hypoxia, which leads to organ ischemia and the development of a pro-inflammatory phenotype. Hypoxic conditions increase neutrophil longevity while increasing endothelial cell permeability, thereby exacerbating vascular leakage [6]. Pulmonary edema and atelectasis heighten lung elastic recoil, reducing lung compliance and alveolar ventilation while disrupting pulmonary blood flow. These changes cause a ventilation-perfusion (V/Q) mismatch [56], marked by ineffective lumen-like ventilation and shunt-like effects, leading to hypoxemia. Research by Ananda S. Mirchandani and colleagues demonstrated that monocyte depletion in a murine hypoxic ALI model resulted in diminished recruitment of monocyte-derived macrophages and exacerbated neutrophil-driven pulmonary inflammation [57]. As a result, tissue hypoxia in the lungs leads to altered immunodynamics and further tissue damage. Moreover, hypoxia induces an oxidant-emphasized response leading to an increase in endogenous reactive oxygen species (ROS) and oxidative responses, and a team from Qinghai University reported that the expression of inflammatory factors, such as IL-17, NF-κB, IL-1β, IL-6, and TNF-α, was significantly elevated after hypoxia [58], which caused further damage to the lung tissue.

## 3. Hypoxia-Inducible Factor (HIF)-1

Oxygen is a vital component for human survival, serving as a key player in metabolic processes. Hypoxia, a condition in which oxygen levels fall below physiological norm, can trigger a cascade of detrimental metabolic events, including cellular apoptosis, inflammation, and tissue injury [59]. HIFs are stabilized under hypoxic conditions and modulate a range of cellular responses and signaling pathways to attenuate the damage caused by hypoxia, including the activation of a range of target genes to promote angiogenesis and metabolic switching [60]. Furthermore, HIF-1 is recognized as a pivotal catalyst in metabolic adaptation to hypoxic conditions [61]. Despite their protective role, HIFs, including HIF-1, can also have a paradoxical effect [62]. While they are instrumental in mitigating the immediate effects of hypoxia, they may simultaneously activate other genetic pathways that could potentially lead to inflammation and contribute to tissue damage.

### 3.1. Activation Mechanisms of HIFs

ALI occurs when localized tissue hypoxia in the lungs is prevalent and has a profound effect on disease progression. Damage to the alveolar epithelium and endothelial cells results in damage to the gas exchange interface and the formation of pulmonary edema, which further impedes the oxygen diffusion process, as well as an imbalance in the ventilation/blood flow ratio. The combination of these factors leads to a significant reduction in the local partial pressure of oxygen in the lungs, creating a hypoxic microenvironment [28]. This hypoxia rapidly and effectively inhibits the enzymatic activity of prolyl hydroxylase (PHD), which blocks the hydroxylation and modification of subunits such as HIF-1α and HIF-2α, thereby reducing their binding to ubiquitin ligases and preventing them from being degraded by the ubiquitin-proteasome system, leading to the accumulation of HIF-α subunits in the cell, which then undergo dimerization and nuclear translocation and combine with HIF-β subunits to form a transcriptionally active complex [63]. For example, in an animal model of lipopolysaccharide (LPS)-induced ALI, the protein expression level of HIF-1α in lung tissues was significantly elevated within a short period of time [64], and its transcriptional activity was correspondingly increased, correlating with the degree of hypoxia, which fully confirms the key driving role of hypoxia in the activation of HIF-1.

In addition to being the core factor of hypoxia, the strong inflammatory response that accompanies ALI also plays an important role in regulating the expression of HIF-1, and the two are intertwined and synergistically influence each other, which together shape the complex expression pattern of HIF-1 in ALI. During the inflammatory response, a variety of inflammatory cytokines, such as TNF–α, IL–1β and IL -6 are released in large quantities. Recent studies have demonstrated that HIF-1α transcription can be activated by these inflammatory cytokines, with a significant effect on HIF-1α expression [65]. For example, TNF-α activates HIF-1α, which is subsequently stabilized by TNF-α under hypoxic conditions, and then transposes to the nucleus and binds to the promoters of target genes, thereby inducing changes in alveolar–capillary barrier permeability [65]. These findings fully demonstrate that hypoxia and inflammatory responses are closely linked through signaling pathways in the pathological process of ALI, together constituting a complex and fine regulatory network, and that hypoxia and inflammatory responses are intertwined to jointly regulate the expression and function of HIF-1.

### 3.2. Structure of HIF-1

**Figure 2:**
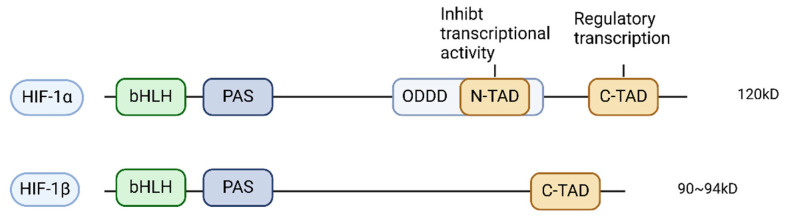
The structure of HIF-1 is depicted in the figure. HIF-1 is a heterodimer consisting of the functional subunit HIF-1α and the structural subunit HIF-1β, with two transcriptionally activated structural domains (TADs), N-TAD, which is a regulator that stabilizes HIF-1α, and C-TAD, which regulates HIF-1α transcription under hypoxic conditions. Created in BioRender. https://BioRender.com.

HIF-1 is a transcriptional activator that functions essentially through oxygen regulation in mammalian development, physiology and disease pathogenesis [66]. HIF-1 is a heterodimer consisting of the functional subunit HIF-1α (120 kD) and the structural subunit HIF-1β (91–94 kD). The HIF-1α subunit is an oxygen-sensitive subunit and the HIF-1β subunit is not sensitive to oxygen [67]. HIF-1α functions as a heterodimeric transcription factor that plays a central regulatory role in the expression of numerous genes responsive to hypoxia and inflammatory stimuli and is essential in regulating immune responses under hypoxia [68]. It modulates cellular responses to hypoxic conditions and diverse environmental cues through direct or indirect regulation of downstream target gene expression [69]. The HIF-1α subunit harbors two transcriptional activation domains (TADs)—the N-terminal (N-TAD) and C-terminal (C-TAD)—both of which are structurally defined by high concentrations of acidic and hydrophobic amino acids, which are functionally recognized as repressor domains. These two transcriptionally activated structural domains are responsible for repressing transcriptional activity under normal oxygen conditions [67]. The C-TAD regulates HIF-1α transcription under hypoxic conditions; in contrast, the N-TAD is a regulator that stabilizes HIF-1α [70] (Figure 2).

### 3.3. Regulatory Mechanisms of HIF-1

**Figure 3:**
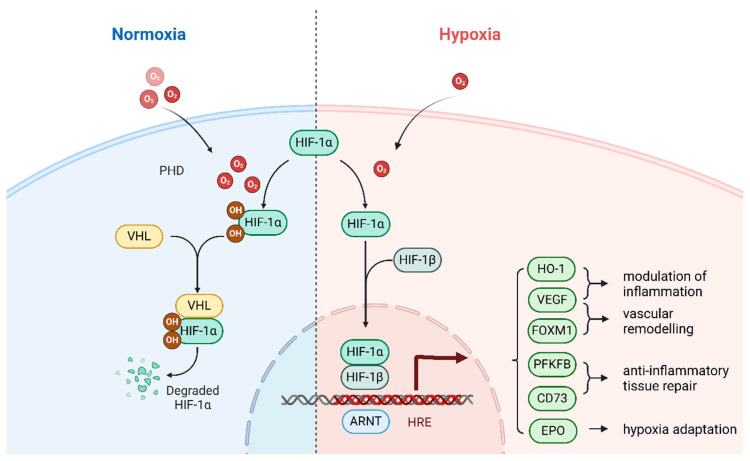
Regulation of HIF-1α under normoxic and hypoxic conditions. Under normal oxygen conditions, HIF-1 is ubiquitinated by PHD and VHL, and under hypoxic conditions, PHD is inactive, HIF-1α stabilizes and migrates from the cytoplasm into the nucleus, subsequently dimerizing with HIF-1β localized in the nucleus to assemble a functional HIF-1 transcriptional complex, which ultimately binds to the HRE in the HIF-1-promoter region to regulate gene expression. Created in BioRender. https://BioRender.com.

Both the stability and transcriptional functionality of HIF-1 are governed by the intracellular oxygen concentration [71], and are core transcription factors that induce the expression of hypoxic genes and repair the intracellular microenvironment. Oxygen- and iron-dependent PHDs hydroxylate proline residues at the HIF-1α subunit under normal oxygen conditions (P402 and P564) [9], thereby facilitating the ubiquitination of HIF-1α and ultimately promoting its degradation via the proteasome. Von Hippel-Lindau (VHL) proteins bind to protein-stabilizing structural domains and play a key role in the ubiquitination of HIF-1α [66].Under hypoxic conditions, the inhibition of PHD activity stabilizes HIF-1α, enabling its translocation from the cytoplasm to the nucleus, where it dimerizes with nuclear HIF-1β to assemble a functional HIF-1 complex. In addition, it ultimately binds to the hypoxia response element (HRE) in the HIF-1-promoter region to regulate genes [72], activate angiogenesis and help cells adapt to hypoxia (Figure 3). The HIF-1 complex functions as a transcriptional activator for numerous genes, including those governing angiogenic pathways such as vascular endothelial growth factor (VEGF), platelet-derived growth factor (PDGF), and angiopoietin-1 (ANGPT1) [73,74]. The HIF-1 complex activates the expression of multiple enzymes critical to anaerobic metabolic pathways, most notably aldolase A and pyruvate kinase. These enzymes are indispensable for the maintenance of basic cellular functions in a low-oxygen environment [74]. The HIF-1α subunit, a key component of the HIF-1 complex, is responsible for activating glycolytic genes, reducing oxygen consumption and mitigating the production of ROS, usually under acute hypoxia [75,76]. It is extremely critical for the protection of cells from oxidative stress damage and helps to maintain cell viability and functional integrity in low oxygen environments. During the complex pathophysiological process of acute injury to the lung, multiple signaling pathways, including the NF-κB [77], Nrf2/HO-1 [78] and NLRP3 [79] pathways, are activated simultaneously in the cell. They regulate the expression of various inflammation-related genes, which in turn affect inflammatory cell activation, chemotaxis and the release of inflammatory mediators, as well as antioxidant defense mechanisms. In contrast, during hypoxia, the main intracellular regulatory mechanism is assumed to involve the HIF-1 signaling pathway [7]. In the complex intracellular signaling network, there is close and clear upstream/downstream regulation between the HIF-1 and PI3K/Akt signaling pathways. The HIF-1 signaling pathway functions as a key downstream component of the PI3K/Akt signaling cascade. The activity of HIF-1α is regulated by the PI3K/Akt/mTOR and PI3K/Akt/FRAP signaling cascades, with activation of the PI3K/Akt/mTOR pathway significantly upregulating HIF-1α expression [80], mTOR is a hypoxia/nutrient sensor and an upstream mediator of HIF-1α activation [81]. In turn, HIF-1α functions as a transcription factor by binding to HREs, thereby inducing the expression of downstream effector genes such as VEGF and erythropoietin (EPO), which initiate adaptive mechanisms to counteract hypoxia, which work synergistically to enable oxygen-tolerant tissues and cells to maintain their normal physiological functions and metabolic homeostasis [82].

## 4. Regulation of HIF-1 in ALI

During ALI, HIF-1α activation exhibits cell type-specific effects: in early phases, it modulates inflammation, vascular leakage, and remodeling in immune/endothelial cells; during progression, HIF-1α activation in ATII cells becomes critical for epithelial proliferation and tissue regeneration [7]. Eckle et al. found that the protective effect of HIF-1α in ALI due to mechanical ventilation was specifically derived from AECs but not from vascular endothelium, myeloid cells or conductive airway epithelial cells by tissue-specific knockout [83]. This suggests a certain spatio-temporal dynamics and tissue specificity of the protective effect of HIF-1α in lung tissue. The discussion below provides a comprehensive overview of the specific effects that result from the activation of HIF-1α in various tissue cells at different stages of ALI.

### 4.1. Dual Role of HIF-1 in ALI-Associated Inflammation

A central pathological feature of ALI/ARDS involves inflammatory dysregulation driven by an excessive influx of inflammatory mediators and immune cells into the interstitial and alveolar compartments of the lung, coupled with disruption of the capillary-alveolar barrier. This cascade culminates in proteinaceous pulmonary edema and consequent hypoxemia [84]. The infiltration of inflammatory cells in lung tissue is therefore a key component in initiating and promoting the inflammatory response associated with ALI, and HIF-1 plays a multifaceted regulatory role in this process. The alveolar epithelium expresses HIF-1α, the specificity of which is characterized by increased enzymatic capacity in ALI, improved mitochondrial respiration and concomitant attenuation of lung inflammation [85]. Hypoxia has been shown to upregulate HIF-1α, which promotes transcriptional repression of the adenosine kinase (AK) [86], thus reducing adenosine from being catalyzed for phosphorylation and extracellular adenosine has been demonstrated to act as a protective modulator of alveolar inflammation in the context of ARDS [87]. Extracellular adenosine signaling via the adenosine A2A receptor (A2AR) increases the level of the immunosuppressive cyclic AMP (cAMP) in immune cells, and cAMP plays a crucial anti-inflammatory role in immune regulation. The anti-inflammatory actions of cAMP are attributed to its ability to modulate pro-catabolic mediators, induce apoptosis and phagocytosis, facilitate non-phagocytic recruitment of macrophages, drive macrophage polarization, and restore elements of tissue homeostasis [88]. These mechanisms collectively mitigate inflammation and tissue damage in vivo [89]. It also reduces pulmonary microvascular permeability and inhibits the secretion of pro-inflammatory cytokines by macrophages [90]. Importantly, the activation of adenosine A2B receptor (A2BR) signaling has been demonstrated to attenuate ALI and confer protective effects in the context of ARDS. Stephen Hatfield et al. reported that HIF-1α is dependent on A2BR-dependent induction of netrin-1 to block neutrophil migration in hypoxic epithelial cells. The hypoxia-adenosine immunosuppressive pathway may be designed to protect tissues from the collateral damage response of overactive immune cells during antipathogen immunity [91]. Among other factors, hypoxia/HIF-1α signaling mediates feedback inhibition of inflammatory processes not only by upregulating ecto-5′-nucleotidase CD73, an enzyme critical for adenosine synthesis, but also by augmenting the expression of adenosine receptors [89]. These findings suggest that the HIF-1α-A2-adenylate signaling pathway, which is activated under low oxygen conditions, mitigates tissue inflammation during immune responses to pathogenic threats [89]. HIF-1α and adenosine receptors may act together to attenuate the efflux of cellular inflammatory factors and attenuate hypoxia-associated vascular leakage and pulmonary edema damage to lung tissue [86,91].

HIF-1α plays a lung-protective role in the induction of the adenosine transcriptional pathway during ALI, suggesting that therapeutically activating the HIF-1α signaling pathway is a potential treatment strategy for ALI/ARDS [7,85,92]. While HIF-1α exerts lung-protective effects through adenosine signaling pathways, its role in inflammation exhibits context-dependent duality. Activation of the mechanistic target of rapamycin (mTOR) signaling pathway may facilitate the translocation of HIF-1α from the cytoplasm to the nucleus, thereby increasing VEGF expression—a process that promotes angiogenesis while increasing vascular permeability, leading to pulmonary edema and damage to the pulmonary capillary endothelium, and an increase in inflammatory factor infiltration [93]. Upon the activation of HIFs, the HIF-1α/hemoglobin oxygenase-1 (HO-1) pathway is subsequently activated and mediates iron death in epithelial cells, whereas iron death can trigger an increase in ROS [94]. Moreover, iron-dead cells release a variety of DAMPs, and ROS may also lead to mitochondrial damage and intracellular redox imbalance, further exacerbating oxidative stress damage [95]. It has also been shown that the HIF-1α pathway is partially dependent on NF-κB signaling in response to TNF-α/IL-4 stimulation in vitro, which in turn induces inflammation-associated cytokine production and chemokines, thereby exacerbating the pathological process of ALI [95,96]. PHD functions as an oxygen-sensing enzyme that modulates HIF-1α activation, and suppression of its enzymatic activity results in the attenuation of inflammatory responses [97]. Moreover, araloside A (ARA) restored the PHD2/HIF-1α axis in pro-inflammatory macrophages, which resulted in the downregulation of HIF-1α expression and the inhibition of pro-inflammatory macrophages [98].

**Figure 4:**
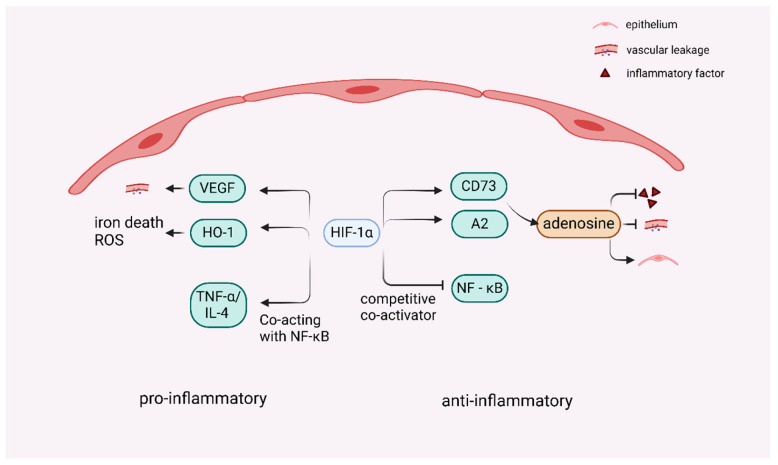
Regulation of inflammation by HIF-1α in ALI. HIF-1α, which is expressed in the alveolar epithelium, exerts anti-inflammatory effects by promoting adenosine production and competing with NF-κB for co-activation, and pro-inflammatory effects by regulating VEGF and HO-1 target genes and co-acting with NF-κB, which increases vascular permeability, leading to the exudation of inflammatory factors as well as the induction of iron death and oxidative stress. Created in BioRender. https://BioRender.com.

HIF-1 also indirectly regulates the release of inflammatory mediators through interactions with other transcription factors. In the complex interaction between HIF-1α and NF-κB, under certain conditions of inflammatory stimuli, HIF-1α can act synergistically with NF-κB [1,99], binding to the regulatory regions of inflammatory mediator genes, promoting their expression, recruiting inflammatory cells such as neutrophils, and releasing inflammatory mediators, thus enhancing inflammatory responses. However, in other cases, HIF-1α can also inhibit NF-κB activity by competing with NF-κB as a coactivator or by modulating its upstream signaling pathway [100], thereby reducing the release of inflammatory mediators. This inter-regulatory relationship forms a fine and complex inflammatory regulatory network that allows the inflammatory response to be regulated within an appropriate range and prevents severe damage to the organism caused by excessive inflammatory responses. Complex crosstalk between HIF-1α and NF-κB occurs in many cell types, and the ultimate outcome is highly dependent on the cell type. In the case of neutrophils, these transcription factors act synergistically, whereas in other cells they antagonize each other [100]. The function of HIF-1α in inflammatory processes is intricate; however, existing evidence indicates that the activation of the HIF-1α pathway predominantly results in anti-inflammatory effects [61]. However, the role of HIF-1α in inflammation and immunity is controversial [101], so it is inferred that whether it exerts pro- or anti-inflammatory effects is related to different cells and the different microenvironments in which it is found (Figure 4).

### 4.2. Regulation of Pulmonary Endothelial Cells and Vascular Remodeling by HIF-1 in ALI

Endothelial cells, key components of the alveolar–capillary membrane, critically influence ALI progression by regulating permeability. HIF-1 modulates vascular permeability through mechanisms that regulate the endothelial cytoskeleton and cell–cell junctions. Endothelial dysfunction--marked by activation and impaired cellular activity--elevates capillary permeability, leading to fluid buildup in alveoli [102], hypoxemia, and aggravated lung damage. However, prolonged or excessive permeability allows protein-rich fluid to flood the interstitial and alveolar spaces of the lung, causing pulmonary edema and amplifying tissue injury. Concurrently with endothelial cell death, repair mechanisms such as angiogenesis (new blood vessel formation) initiate endothelial layer reconstruction. Angiogenesis is vital during ALI recovery, restoring lung structure and function as a primary endogenous repair process. The endothelial network, which spans capillaries and larger blood vessels, acts as a metabolically active barrier. It governs vascular tone, immune cell movement, and remodeling [103]. During remodeling, angiogenesis involves the formation of new blood vessels driven by the migration and proliferation of endothelial cells from established vascular structures [104].

In addition to the neovascularization and remodeling described above, it has been proposed that HIF-1α significantly contributes to vascular repair and the resolution of inflammation in patients with ARDS via the forkhead box protein M1 (FOXM1) signaling pathway in endothelial cells during the tissue repair phase after lung injury [75]. HIF-1α-inhibited mice exhibit impaired vascular repair, a sustained inflammatory response and increased mortality. Thus these studies provide evidence that endothelial HIF-1α-Foxm1 signaling serves as a crucial endogenous mechanism for vascular restoration following inflammatory damage [105], and is a powerful potential target and signaling pathway for the treatment of ALI. As angiogenesis progresses, the extracellular matrix surrounding the vessels changes accordingly, providing an optimal microenvironment for alveolar cell regeneration and migration. Moreover, HIF-1α modulates the proliferation and differentiation processes of vascular smooth muscle cells, affects the thickness and elasticity of the vascular wall, and promotes the maturation and stabilization of blood vessels to adapt to the functional needs of repaired lung tissue.

Furthermore, HIF-1α acts as a transcription factor that regulates the expression of downstream target genes, including VEGF, EPO and iNOS, and induces the expression of proteins involved in glycolysis, angiogenesis and cell survival, thereby promoting cellular adaptation to hypoxia [106]. Moreover, previous studies have shown that tissue hypoxia is a potent inducer of VEGF expression in multiple organs [107]. In the hypoxic environment of ALI, HIF-1 is activated, and the stability of the HIF-1α subunit is increased, which then transfers to the nucleus where it binds to HIF-1β to form an active dimer [108]. This dimer binds to the HRE of the VEGF gene and promotes VEGF transcription and expression. Thus the strong upregulation of VEGF mediated by the transcription factor HIF-1 [107]. VEGF is an important angiogenic factor that stimulates the proliferation and migration of endothelial cells. Research from Capital Medical University revealed that the PI3K-Akt-mTOR signaling pathway may be activated in pulmonary artery endothelial cells (PAECs) under hypoxic conditions, promoting pulmonary vascular remodeling [109]. Previous reports have shown that PI3K/AKT phosphorylation induces an increase in HIF-1α transcription under hypoxic conditions, and that HIF-1 is a central regulator of angiogenesis postischemia [110]. Following lung injury, extensive leakage of plasma proteins and activation of inflammatory cells occur, followed by relative lung ischemia and hypoxia. Throughout this process, substantial quantities of HIF-1α are synthesized [110]. HIF-1α targets VEGF, and HIF-1α can interact with the promoter region of the VEGF gene or related regulatory elements through a series of complex molecular mechanisms to promote VEGF expression and synthesis. When VEGF is secreted, it binds to the vascular endothelial growth factor receptor 2 (VEGFR2) located on the endothelial cell surface, thereby facilitating the activation of the autocrine VEGF/VEGFR2 signaling loop within endothelial cells. Therefore, the HIF-1α/VEGF signaling pathway plays a crucial role in tissue angiogenesis, providing the necessary cellular basis and molecular signaling support for the formation of new blood vessels, and facilitating angiogenesis in tissues [111]. Angiogenesis facilitates the restoration of the alveolar epithelial-endothelial barrier, decreases the extravasation of cytokines and inflammatory cells, and may improve ischemia in lung tissue. The HIF1α-mediated increase in VEGF expression in lung tissue stimulates endothelial cell proliferation, migration and lumen formation and enhances vessel density. This phenomenon improves the microcirculation of the lung tissue, reduces tissue hypoxia, helps reduce endothelial cell damage and promotes their repair, which in turn reduces vascular permeability to a certain extent and restores the normal function of the alveolar–capillary membrane to help promote ALI repair.

### 4.3. Regulation of Lung Epithelial Cells and Lung Macrophages by HIF-1 in ALI

ALI is defined by respiratory failure resulting from disruption of the pulmonary epithelial and endothelial barriers. This disturbance results in the accumulation of protein-rich fluid within the alveolar spaces and the influx of neutrophils into the alveolar region [112]. Diffuse lung epithelial cell injury is one of the major mechanisms driving ALI [113]. During hypoxia, HIF-1α facilitates cellular adaptation to environments with diminished oxygen availability [96]. HIF-1α is a modulator of carbohydrate metabolism in AECs during ALI. Studies of ALI have shown that HIF-1α stabilizes and contributes to the optimization of alveolar-epithelial carbohydrate metabolism in vitro or in vivo models of alveolar injury, and that the absence of HIF-1α in the alveolar-epithelium indicates that lung injury is exacerbated during exposure to ARDS [87]. These findings suggest that HIF-1α and the glycolytic pathway are crucially involved in ALI. The AECs are flat type I and cuboidal type II, which work closely together to maintain the normal structure and function of the alveoli. In particular, alveolar epithelial barrier function is essential for preventing leakage of fluid and proteins from the alveoli and maintaining alveolar gas exchange. HIF-1 preserves the integrity of the alveolar epithelial barrier in AECs through the regulation of numerous genes associated with barrier function. Among them, ATII cells serve a critical function in the repair of ALI [114]. The progenitor cell properties of ATII cells indicate their outstanding potential for treating airway diseases [115]. ATII cells exhibit enhanced glycolysis via the HIF1α-phosphofructokinase-2/fructose-2,6-bisphosphatase (PFKFB) 3 axis, which enhances glycolysis and releases the product of glycolysis, lactate. Lung macrophages shift from a pro-inflammatory phenotype to an anti-inflammatory phenotype and increase the levels of the anti-inflammatory markers IL-10 and Arginase-1 (Arg-1) in the microenvironment of lactic acid produced by ATII cells [116]. Therefore, PFKFB3 in the alveolar epithelium plays a crucial role in enhancing the integrity of the alveoli by increasing glycolytic carbohydrate metabolism during ALI [114], which is important for recovery from ALI. Stromal cell derived factor (SDF)1 and its receptor CXCR4 are target genes in ATII cells, and the upregulation of HIF-1α during lung injury enhances ATII cells migration in vitro and facilitates the restoration of barrier function in vivo [7]. Thus epithelial HIF-1α has also been shown to be important in promoting alveolar proliferation and SDF1 and its receptor chemokine, CXCR4, are expressed in stromal cells via HIF-1α targets [117].

The ability of AECs to proliferate and repair is critical for the recovery of lung tissue after ALI, and HIF-1 plays an active role in facilitating this process. HIF-1α stimulates the proliferation and differentiation of ATII cells by upregulating the expression of some growth factors and increasing glycolysis, thereby promoting alveolar epithelial repair. However, elevated levels of HIF-1α expression coincide with pathological damage to AECs. HIF-1α has been shown to participate in ATP synthesis, cell proliferation, and apoptosis in AECs. Under hypoxic conditions, HIF-1α enhances glycolytic activity and acidifies the extracellular environment, which subsequently promotes apoptosis [118]. This is because the effect of lactic acid depends on the specific lung compartment; in direct ALI, lactic acid is protective, whereas in indirect ALI, the inhibition of glycolysis attenuates lung injury [116]. Additionally, sustained high levels of HIF-1α over a long period of time may lead to the development of pulmonary fibrosis (PF). The development of PF is usually primarily the result of prior acute lung inflammation [119]. PF after ALI is a process in which inflammation in the lungs leads to persistent alveolar damage, extracellular matrix production, repetitive cell destruction, repair and reconstitution, and excessive collagen deposition. Under hypoxic conditions, AECs can increase the level of ROS in mitochondria, and ROS can up-regulate the expression of HIF-1α and TGF-β 1 to promote the progression of epithelial–mesenchymal transition (EMT) [82]. It has been demonstrated that paraquat-induced PF can be attenuated by down-regulating the HIF-1α/β-catenin pathway and inhibiting the EMT [82,120]. One of the many key causes of genetic changes in the disease is high levels of HIF-1α, and researchers have found that patients with idiopathic pulmonary fibrosis have abnormally elevated levels of lactic acid in the lungs and serum [121]. It was mentioned above that HIF-1α can release lactate via glycolysis, which converts macrophages to an anti-inflammatory phenotype. However, the accumulation of lactate also activates TGF-β1 in a pH-dependent manner in vitro, thereby inducing differentiation of lung fibroblasts. CCT6A is a member of the chaperone-containing TCP1 complex (CCT), which reduces lung fibrosis by inhibiting HIF-1α protein levels and lowering lactate [121]. Therefore, in the late stages of ALI and tissue repair, the expression level of HIF-1α and lung injury should be detected in a timely manner, and active intervention should be carried out in order to avoid leading to PF.

### 4.4. Regulation of Cell and Lung Tissue Repair by HIF-1 in ALI

Cell death including apoptosis and programmed cell death, which are associated with inflammation and ischemia–reperfusion injury, is also a key issue in ALI [122]. Apoptosis in ALI is controlled mainly by exogenous pathways mediated by cell surface death receptors, and by endogenous pathways mediated by mitochondria, which cause apoptosis in AECs via stimuli that trigger death induction and DNA damage [123]. Programmed cell death in ALI is triggered by activated pathways in which lipid peroxidation causes iron death through mitochondrial contraction. Iron death primarily results from impaired membrane lipid repair mechanisms, which subsequently cause an accumulation of ROS on membrane lipids, and excessive buildup of ROS further leads to oxidative damage and inflammatory responses within lung tissue [122].

As a result, damage to and death of epithelial cells leads to disruption of the epithelial-endothelial barrier, which in turn leads to increased vascular permeability and leakage of proteins, inflammatory factors, etc., resulting in damage to lung tissue. Therefore the treatment of lung injury also focuses on the repair of cells and lung tissue. HIF-1α affects ALI by participating in the regulation of inflammation, cell proliferation and immune responses [124]. HIF-1α increases adenosine signaling induced through Adora2a adenosine receptors. Enabling adenosine receptors to produce adenosine extracellularly and signaling through adenosine receptors has a beneficial role in lung protection, e.g., by attenuating vascular leakage or improving alveolar fluid transport [83]. Additionally, adenosine is highly anti-inflammatory and reduces cytokine and leukocyte release, thus limiting leukocyte extravasation and the immune response [125]. Therefore, HIF-1α appears to play a role in promoting tissue regeneration and modulating cellular tolerance mechanisms [96]. It has also been shown that HIF-1α promotes neutrophil survival and increases phagocytosis, which should be beneficial to the host during bacterial or viral infections [126]. HIF-1α also prolongs the lifespan of neutrophils by inhibiting apoptotic signaling and increases their antimicrobial function by upregulating the expression of related molecules [55]. HIF-mediated enhancement of the chemokine SDF1 and its receptor CXCR4 and the activation of the SDF1/CXCR4 signaling pathway facilitate the spreading of ATII cells and accelerate the recovery of the alveolar barrier [127]. The promotion of ATII cells proliferation by HIF-1α may also be associated with the increased expression of various growth factors, glucose transporters, and glycolytic enzymes, which subsequently drive the synthesis of macromolecules (such as nucleic acids, lipids, and proteins) necessary for cell replication [7]. HIF-1α therefore promotes the repair of adjacent surviving cells and tissues by upregulating different target genes (e.g., VEGF, CXCR4 and SDF1).

### 4.5. Potential Influencing Factors and Drug Study of Targeting HIF-1 for ALI Treatment

The clinical classification of ALI and ARDS into direct (e.g., pneumonia) or indirect (e.g., blood transfusion, pancreatitis) clinical predictors and their biomarker profiles differed as mentioned previously; however, there is currently no evidence of treatment differences on this basis [128,129,130]. The prediction of ARDS in critically ill patients without ARDS on admission may be enhanced by plasma biomarkers such as angiopoietin-2 [130,131]. Developing pharmacological prophylaxis requires deeper insight into how individual biological variability influences clinical lung injury risk. Key objectives in drug discovery encompass enhancing mechanical lung injury protection, promoting lung repair and fibroplasia regression, and inhibiting ‘biotrauma’-mediated extrapulmonary organ failure [131]. Studies of drugs targeting alveolar epithelial and vascular endothelial damage may be limited to direct and indirect sources of ARDS, respectively [131]. In a similar manner, disparities in ALI and patient heterogeneity resulting from diverse factors may result in differential activation and functioning of the HIF-1α pathway. It is therefore necessary to take these factors into account in the subsequent targeting research process as well as in clinical trials. For instance, high blood glucose levels can lead to chronic inflammation and oxidative stress, thereby increasing the production of various pro-inflammatory cytokines [132]. Therefore, it is hypothesized that high blood glucose in patients who are originally diabetic may affect the stability of HIF-1α and the regulation of ALI. Global gene expression analysis reveals significant differences between men and women in exposure to many diseases. In asthma, the HIF-1 signaling pathway is enriched in males and the chemokine signaling pathway is enriched in females [133]. As a result, it remains to be investigated whether HIF-1 is also gender-specific in ALI. Whether the ultimate effect of HIF-1α is to exacerbate injury or provide protection (or both) is highly dependent on the unique genetic, physiological and pathological background of the individual patient. A deeper understanding of this patient-specific relationship has an important role to play in achieving the diagnostic and therapeutic of ALI/ARDS.

The study and discussion of a substantial corpus of literature reveals HIF-1α to be an intricate and dual-acting regulator in ALI, characterized by dual-acting properties. Its protective effect is dependent on the type of injury, stage and microenvironment. Therefore, how to precisely regulate is a central challenge for future research and clinical translation. Drugs targeting HIF-1α that have been used experimentally or in mouse model include Emodin (ameliorates LPS-induced pathological changes in ALI and significantly reduces the expression of various inflammatory factors through the mTOR/HIF-1α/VEGF signaling pathway) [82,93], Emodin (reduces paraquat-induced ALI and PF) [82,120], COMP-Ang1 (significantly attenuates lung injury by inhibiting HIF-1α in a mouse model of hydrogen peroxide (H_2_O_2_)-induced ALI) [82,134], DMOG (HIF-1α stabilizer, protective against LPS-induced lung injury through HIF-1α-dependent glycolysis enhancement) [135], Roxadustat (inhibits respiratory virus-induced lung epithelial cell damage through HIF-1α dependence) [136], etc. Of these drugs, Roxadustat has been approved for clinical use in several countries as an erythropoiesis stimulator for patients with anemia and chronic kidney disease. It is currently used in dialysis [136]. Although no HIF-1α-targeting agent has advanced to ALI clinical trials, these findings provide critical guidance for developing targeted therapies. Strategic selection of such agents requires analysis of injury etiology and ALI stage to determine whether HIF-1α stabilization or suppression is indicated, enabling personalized therapeutic approaches.

## 5. Discussion

ALI is a process characterized by increased permeability, pulmonary edema, hypoxemia and severe inflammation of the lungs due to a variety of direct and indirect factors, usually resulting in a high mortality rate [137]. It is characterized by acute inflammation, endothelial barrier disruption and alveolar epithelial damage, resulting in edema of the protein—abundant pulmonary interstitium and leakage of immune cells into the alveolar lumen. Therefore, promoting the abatement of inflammation in the lungs and facilitating lung repair is a therapeutic strategy for potential ALI/ARDS [138].

The clinical syndrome of ALI is characterized by acute lung inflammation [139], i.e., the acute phase in which the injury occurs is predominantly inflammatory, and ALI and inflammation are interdependent, forming a vicious cycle that may lead to irreversible damage [94]. Understanding the mechanisms of inflammation and controlling it in a timely manner is therefore important in arresting the progression of lung injury. Research has demonstrated that signaling pathways dependent on hypoxia are linked to a reduction in mucosal inflammation [83] and that HIF-1α exerts anti-inflammatory effects. As detailed above, existing studies have shown that the HIF pathway is activated under hypoxic conditions and that transcription factors such as HIF-1 can regulate the expression of genes related to adenosine metabolism. For example, the expression of extracellular nucleosidases (e.g., CD73) that catalyze the generation of adenosine from AMP can be upregulated, increasing the concentration of extracellular adenosine. Adenosine is widely recognized as a protective and anti-inflammatory agent because of its ability to inhibit signaling pathways essential for the production and release of pro-inflammatory cytokines. It also mitigates inflammatory processes and promotes cell protection by enhancing oxygen delivery, triggering anti-inflammatory responses, inducing preconditioning effects, and stimulating angiogenesis [140]. After the inhibition of PHD activity, the expression of HIF-1α in the lungs increases through the HIF-1α/HO-1 signaling pathway. Its ability to increase survival, reduce inflammation, inhibit oxidative stress and regulate mitochondrial dynamic homeostasis ameliorates LPS-induced lung injury and inflammation [75,141,142], thus moderate overexpression of HIF-1α was demonstrated to have an anti-inflammatory effect [143]. However, as mentioned above, activation of the HIF-1α/HO-1 pathway has been shown to induce iron death and ROS accumulation [144]. Activation of the HIF-1α/VEGF pathway also increases vascular permeability leading to exudation and infiltration of inflammatory factors [93,145], thus exacerbating the inflammatory response. In addition to these direct regulatory mechanisms, HIF-1α can also be regulated through interactions with other transcription factors, such as through coaction with NF-κB and indirect regulation of inflammatory mediator release. A review of the literature revealed that HIF-1α has both anti-inflammatory and pro-inflammatory effects on ALI. As a result, this difference is considered to be due to a different microenvironment in terms of what induced the ALI and the resulting ALI. Under different pathological conditions, HIF-1α signaling can lead to pro-inflammatory or anti-inflammatory effects through direct or indirect pathways [105], and further studies are needed to confirm this finding.

The characteristics of ALI include the unregulated infiltration of inflammatory cells into various pulmonary compartments, the secretion and activation of inflammatory mediators including cytokines, and the presence of severe arterial hypoxemia. In the early stages of ALI, the most critical and primary focus is on effective control of the inflammatory response. As the overactivation of inflammation during this period is often an important factor leading to further deterioration of the disease, the core of the work should be decisively focused on the repair of the lung tissue as the treatment process advances into the later stages of the disease. Lung tissue may suffer varying degrees of damage due to the effects of pre-inflammation, such as destruction of alveolar structures and fibrotic changes in the interstitial tissue. At this point, it is necessary to gradually repair damaged lung tissue, restore its normal physiological function and structural integrity, and thus achieve comprehensive improvement and rehabilitation of the patient’s lung function.

In the pathogenesis of ALI, alveolar epithelial and endothelial damage plays a key role, leading to disruption of the alveolar capillary barrier, widespread pulmonary edema, reduced gas exchange and uncontrolled lung inflammation. [83]. The natural barrier constituted by AECs and vascular endothelial cells serves to prevent proteins and other substances from blood vessels from infiltrating the alveoli and thus causing edema. However, during ALI, the natural barriers that make up these cells are attacked and disrupted, leading to the apoptosis or autophagy of AECs and vascular endothelial cells which subsequently results in elevated vascular permeability [42,146,147], and affects lung ventilation, thereby triggering hypoxemia and the upregulation of HIFs. HIF-1α, in turn, induces the expression of pro-apoptotic genes in susceptible cells by upregulating the expression of different target genes (e.g., VEGF, CXCR4, and SDF1) while promoting the repair of surrounding surviving cells [7]. As detailed above, both the HIF-1α-Foxm1 and HIF1α/VEGF signaling pathways promote increased proliferation of lung endothelial cells and vascular repair [148,149]. Endothelial cells begin to gradually build new vascular structures, and the newly formed blood vessels are able to supply oxygenated blood precisely to specific areas of lung tissue [149]. Improving ischemia in lung tissue effectively due to ALI. After ischemic and hypoxic conditions are improved, lung tissue cells can repair and regenerate themselves in a better microenvironment, and the damaged cell structure and function are gradually restored, which strongly promotes the repair process of the lung tissue as a whole, and ultimately creates extremely favorable conditions for the healing of ALI.

**Figure 5:**
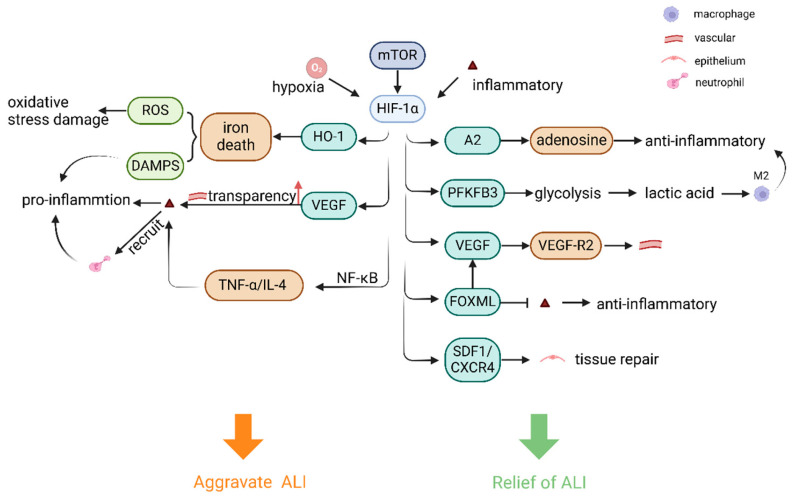
Regulation of ALI by HIF-1. HIF-1 plays a dual role in ALI by regulating different target genes. The left part of the figure shows that HIF-1α contributes to the development of inflammation and oxidative stress and exacerbates ALI by regulating HO-1 and VEGF, and cooperatively interacts with NF-κB, whereas the right part of the figure shows that HIF-1α contributes to the development of ALI through the regulation of glycolysis, adenosine, the FOXM1 signaling pathway, and the angiogenic factor VEGF, as well as alveolar proliferation and SDF1, and promotes angiogenesis and tissue repair through the regulation of receptor CXCR4 expression, which is important for the alleviation of ALI. Created in BioRender. https://BioRender.com.

HIF-1α plays an extremely critical role in the complex pathophysiological processes of ALI (Figure 5). On the one hand, HIF-1α promotes endothelial cell proliferation and induces the upregulation of the expression of key angiogenesis-related factors, such as VEGF, through a series of cascade reactions, thereby stimulating endothelial cells to migrate, differentiate, and form new vascular structures, and improving blood perfusion in lung tissue. In addition, HIF-1α is a transcription factor that regulates lung epithelial cells in ALI. Following acute inflammation in ALI, the lung undergoes repair and remodeling. The alveolar epithelium plays a key role in protecting the lungs from infectious agents and ATII cells play crucial secretory and regenerative functions in the alveoli to preserve pulmonary homeostasis [139,150,151]. As mentioned above, by activating the HIF-1α/PFKFB pathway, which enhances glycolysis in lung epithelial cells and leads to the production of lactic acid by epithelial cells, pro-inflammatory macrophages can be induced to transform into anti-inflammatory macrophages [152]. Emerging research increasingly indicates that alterations in metabolic pathways and signaling mechanisms involving metabolic intermediates are crucial in shaping the AM phenotype [116,153] and that promoting glycolytic metabolism in ischemic tissues improves cell survival [154]. An increase in lactate and anti-inflammatory macrophages reduces epithelial inflammation and leads to the maturation and functional strengthening of ATII cells, which is essential for maintaining the secretion of alveolar surface-active substances [151] and the stability of the alveolar structure. ATII cell, as a progenitor of ATI cell, activates the repair program after lung injury and proliferates and differentiates into ATI cell cells, which migrate to the damaged area and participate in the repair of the damaged alveolar epithelium [155,156]. Recognized mechanisms for the maintenance and repair of alveoli involve stem cells that possess the ability to self-renew over extended periods. The upregulation of HIF-1α promotes the expression of alveolar proliferation and SDF1 and its receptor CXCR4 in stromal cells to mediate cell proliferation, survival and migration [157], which in turn contributes to the repair of lung tissue. A2B adenosine, which is mediated by HIF-1α as mentioned above, not only plays an important role in inflammation, but also improves lung injury and alveolar fluid clearance during ALI, thus alveolar epithelial A2B adenosine receptors contribute to protective mechanisms in the lung during ALI [158].

Hypoxia can initially be an inflammatory stimulus that promotes a pro-inflammatory response and disrupts the tissue barrier [159,160], at which point HIF-1 can stabilize to the point where it regulates the expression of a number of genes, promoting cell adaptation and survival [161]. The enhancement of the extracellular adenosine pathway during ARDS via HIF-1α can be enhanced in a number of ways, including the induction of extracellular 5′-nucleosidase (conversion of extracellular AMP to adenosine) via the transcriptional enzyme production of adenosine, a known HIF-1α target gene [83] to increase protection against the effects of extracellular adenosine signaling during ALI to promote cell and tissue repair. Increased extracellular adenosine not only has an anti-inflammatory effect but also has a role in the repair of lung epithelium and endothelium as well as blood vessels. Through these mechanisms, HIF-1α attenuates both vascular leakage and inflammation by modulating A2A adenosine receptors, and previous studies have also established that HIF-1α modulates A2B adenosine receptors during ARDS [162]. Functional studies of A2B signaling during ARDS suggest that the role of A2B adenosine receptors in improving alveolar fluid clearance and attenuating lung injury occurs through the induction of mechanical ventilation [162]. Thus, in acute injury, adenosine signaling plays an anti-inflammatory, tissue-protective role, particularly through the activation of adenosine 2A and 2B receptors [163]. However, excessively chronic adenosine promotes pro-inflammatory, tissue destruction [163], and studies have shown that a potentially elevated adenosine level has a definite deleterious effect on chronic lung disease [162]. Therefore, targeting the adenosine pathway HIF-adenosine via HIF-1α under hypoxia is feasible for the treatment of ALI.

HIF-1α plays an important role as described above, and it has also been demonstrated that HIF-2α shares target co-operation with HIF-1α, but there are also differences, and is equally a target that can be considered for ALI treatment. HIF-1α and HIF-2α are core transcription factors for cellular sensing and response to hypoxic environments, share core oxygen sensing and transcriptional activation mechanisms, and co-regulate many important adaptive genes. They play a key role in both the normal physiological function of the lungs and in the pathology of a variety of lung injuries (e.g., ALI/ARDS, chronic obstructive pulmonary disease, PF, pulmonary hypertension). Both HIF-1α and HIF-2α are multifunctional and are associated with causing ARDS severity. HIF-1α is generally induced more rapidly and is more prominent in driving intense inflammatory responses, metabolic shifts, and early increases in vascular permeability in the acute phase; HIF-2α, on the other hand, is delayed induction [164], and HIF-2α typically plays a more critical and specific role in chronic adaptation as well as functional maintenance of specific cells (e.g., endothelium, ATII), and promotion of fibrosis. HIF-2α is associated with cell differentiation and regeneration [165] and its stabilization in ATII cells contributes to the maturation of type II cells and to the regulation of genes that encode and are involved in important proteins involved in surfactant production, and can therefore be considered essential for lung repair and regeneration after injury [166].In addition, HIF-1α appeared to be universally expressed, whereas HIF-2α was detected in a more restricted set of cell types, including vascular endothelial cells, ATII cells, and macrophages [167]. It has recently been shown that the role of HIF-2α in enhancing endothelial barrier integrity and thus inhibiting vascular damage in response to septic attack [105]. Thus HIF-1α has an important role in the pre-inflammatory regulation of ALI, whereas in the later stages of lung tissue repair, HIF-1α and HIF-2α could be considered as common targets to be considered to promote repair.

In ALI, HIF-1 modulates inflammation, epithelial/endothelial repair, and vascular regeneration under hypoxic conditions, offering protective effects against lung damage. However, prolonged hypoxia and sustained HIF-1 activation exacerbate injury by increasing inflammation, inducing alveolar epithelial cell apoptosis, and worsening tissue destruction or induce PF. These dual roles highlight the need for timely oxygen therapy and pharmacological interventions to mitigate pathological outcomes while preserving the reparative functions of HIF-1. And it is important to consider factors such as patient heterogeneity and the stage of progression of the ALI, as this will help to ensure that the correct treatment is administered.

## 6. Conclusions and Outlook

ALI can arise from multiple causes, leading to diminished lung volume, impaired compliance, disruptions in ventilation/blood imbalance, and hypoxemia due to its complex pathophysiology including lung inflammation, increased alveolar permeability and damage to the alveolar epithelium and vascular endothelium [168]. Hypoxia is a condition in which low or insufficient levels of oxygen are present in tissues and is associated with physiological and pathological conditions. The main regulator that responds to this situation to mediate cellular activity is HIF-1 [62]. Inflammation, macrophages, neutrophils, leukocytes, and lung endothelial and epithelial cells in ALI are modulated by HIF-1. HIF-1 attenuates inflammation through regulation of the downstream target adenosine; modulation of the target genes Foxm1, VEGF, CXCR4 and SDF1 and adenosine promotes the transformation of lung macrophages into anti-inflammatory cells and facilitates the reconstruction of blood vessels and the repair of the pulmonary endothelial–epithelial barrier, which is important for the repair of lung injury.

In many cases, endogenous pathways contribute to the regression of different lung injuries by controlling excessive lung inflammation. HIF-1 activation can be protective under hypoxic microenvironmental conditions [62]. However, the regulatory role of HIF-1 has two sides; for example, while activating VEGF to promote revascularization, this factor can exacerbate inflammation by promoting the leakage of inflammatory factors and proteins as it is also a permeability increasing factor [169]. The downstream SDF1/CXCR4 axis also converges and accumulates inflammatory cells (neutrophils, monocytes and lymphocytes) into local tissues and regulates the release of inflammatory factors that elicit an inflammatory response [170] and is associated with late lung fibrosis [171,172].

In this review, hypoxia was identified as the principal mechanism through which HIF-1 exerts dual regulatory effects on ALI. Hypoxic signaling and HIF-1 activation are discussed in terms of their bidirectional impact on inflammation and lung tissue repair. The HIF-1 signaling pathway is a significant factor in the pathogenesis of ALI and inflammation and represents a promising therapeutic target for ALI treatment. However, the role of HIF-1 in ALI remains a subject of debate and warrants further investigation. Research has been conducted to develop drugs that modulate HIF-1 activity, either by stabilizing the HIF-1α protein or by influencing its transcriptional activity. These potential therapeutic agents show promise for the treatment of ALI, presenting new clinical care options. Given that HIF-1 activation is a complex process involving multiple signaling pathways, a key challenge is to precisely regulate HIF-1 activity within the complex pathophysiological context of ALI, thereby avoiding overactivation and its associated adverse effects.

## Data Availability

No new data were created or analyzed in this study. Data sharing is not applicable to this article.

## Abbreviations

The following abbreviations are used in this manuscript:

ALI	Acute lung injury
HIF	Hypoxia-inducible factor
ARDS	Acute respiratory distress syndrome
LECs	Lymphatic endothelial cells
AECs	Alveolar epithelial cells
GM-CSF	Granulocyte-macrophage stimulator
TBI	Traumatic brain injury
BRMs	Biological response modifiers
DAMPs	Damage-associated molecules
PRRs	Pattern recognition receptors
ATI cell	Alveolar type I epithelial cell
ATII cell	Alveolar type II epithelial cell
TNF-α	Tumor necrosis factor-alpha
IL-6	Interleukin 6
IL-1β	Interleukin-1β
IL-12	Interleukin 12
IL-10	Interleukin 10
AMs	Alveolar macrophages
IFN-γ	Interferon-γ
LPS	Lipopolysaccharide
iNOS	Inducible nitric oxide synthase
ROS	Reactive oxygen species
V/Q	ventilation-perfusion
PHD	Prolyl hydroxylase
VHL	Von Hippel-Lindau
HRE	Hypoxia response element
TADs	Transcriptional activation domains
VEGF	Vascular endothelial growth factor
PDGF	Platelet-derived growth factor
PI3K	Phosphatidylinositol 3-kinase
Akt	Protein kinase B
mTOR	mechanistic Target Of Rapamycin
A2AR	Adenosine A2A receptor
cAMP	cyclic AMP
A2BR	Adenosine A2B receptor
CD73	Cluster of differentiation 73, ecto-5′-nucleotidase
HO-1	Hemoglobin oxygenase-1
ARA	Araloside A
FOXM1	Forkhead box protein M1
PAECs	Pulmonary artery endothelial cells
VEGFR2	Endothelial growth factor receptor 2
PFKFB	Phosphofructokinase-2/fructose-2,6-bisphosphatase
Arg-1	Arginase-1
SDF	Stromal cell derived factor
CXCR4	C-X-C chemokine receptor type 4
PF	Pulmonary fibrosis
EMT	Epithelial–mesenchymal transition
TGF-β1	Transforming growth factor beta 1
CCT6A	A member of the chaperone-containing TCP1 complex (CCT)
H_2_O_2_	Hydrogen peroxide
